# Lycopene Supplementation to Serum-Free Maturation Medium Improves In Vitro Bovine Embryo Development and Quality and Modulates Embryonic Transcriptomic Profile

**DOI:** 10.3390/antiox11020344

**Published:** 2022-02-10

**Authors:** Shehu Sidi, Osvaldo Bogado Pascottini, Daniel Angel-Velez, Nima Azari-Dolatabad, Krishna Chaitanya Pavani, Gretania Residiwati, Tim Meese, Filip Van Nieuwerburgh, Elias Kambai Bawa, Ambrose Alikidon Voh, Joseph Olusegun Ayo, Ann Van Soom

**Affiliations:** 1 Department of Internal Medicine, Reproduction and Population Medicine, Faculty of Veterinary Medicine, Ghent University, 9820 Merelbeke, Belgium; shehu.sidi@udusok.edu.ng (S.S.); daniel.angelvelez@ugent.be (D.A.-V.); nima.azaridolatabad@ugent.be (N.A.-D.); krishnachaitanya.pavani@ugent.be (K.C.P.); habibresibismillah@gmail.com (G.R.); ann.vansoom@ugent.be (A.V.S.); 2 Department of Theriogenology and Animal Production, Usmanu Danfodiyo University, Sokoto 840231, Nigeria; 3 Gamete Research Center, Laboratory for Veterinary Physiology and Biochemistry, Department of Veterinary Sciences, University of Antwerp, 2610 Wilrijk, Belgium; 4 Research Group in Animal Sciences—INCA-CES, Universidad CES, Medellin 050021, Colombia; 5 Laboratory for Pharmaceutical Biotechnology, Faculty of Pharmaceutical Science, Ghent University, 9000 Ghent, Belgium; tim.meese@ugent.be (T.M.); filip.vannieuwerburgh@ugent.be (F.V.N.); 6 National Animal Production Research Institute, Zaria 810241, Nigeria; ekbawa@abu.edu.ng (E.K.B.); aavoh@abu.edu.ng (A.A.V.); 7 Department of Veterinary Physiology, Ahmadu Bello University, Zaria 810006, Nigeria; joayo@abu.edu.ng

**Keywords:** antioxidant, embryo production, embryo quality, reactive oxygen species, RNA-seq, vitrification

## Abstract

Bovine embryos are typically cultured at reduced oxygen tension to lower the impact of oxidative stress on embryo development. However, oocyte in vitro maturation (IVM) is performed at atmospheric oxygen tension since low oxygen during maturation has a negative impact on oocyte developmental competence. Lycopene, a carotenoid, acts as a powerful antioxidant and may protect the oocyte against oxidative stress during maturation at atmospheric oxygen conditions. Here, we assessed the effect of adding 0.2 μM lycopene (antioxidant), 5 μM menadione (pro-oxidant), and their combination on the generation of reactive oxygen species (ROS) in matured oocytes and the subsequent development, quality, and transcriptome of the blastocysts in a bovine in vitro model. ROS fluorescent intensity in matured oocytes was significantly lower in the lycopene group, and the resulting embryos showed a significantly higher blastocyst rate on day 8 and a lower apoptotic cell ratio than all other groups. Transcriptomic analysis disclosed a total of 296 differentially expressed genes (Benjamini–Hochberg-adjusted *p* < 0.05 and ≥ 1-log2-fold change) between the lycopene and control groups, where pathways associated with cellular function, metabolism, DNA repair, and anti-apoptosis were upregulated in the lycopene group. Lycopene supplementation to serum-free maturation medium neutralized excess ROS during maturation, enhanced blastocyst development and quality, and modulated the transcriptomic landscape.

## 1. Introduction

Reactive oxygen species (ROS) are reactive molecules with high pro-oxidative potential that are responsible for oxidative stress [1]. Despite the negative connotation associated with oxidative stress, ROS are also required in physiological reproductive processes, playing a significant role in sperm capacitation and fertilization [2,3]. During in vivo conditions, the presence of intrinsic and extrinsic antioxidants within the ovarian follicle neutralize any excess ROS, maintaining the equilibrium between a pro- and anti-oxidative milieu [4]. In contrast, during in vitro embryo production (IVEP), it is challenging to recreate a balanced oxidative environment due to the high oxygen tensions to which the oocytes are exposed during manipulation and in vitro maturation (IVM).

IVM is routinely performed under 5% carbon dioxide (CO_2_) and 21% oxygen (O_2_) in air [5], which is higher than the conditions that prevail within the ovarian follicle [6]. Hence, greater oxygen tension during IVM may increase ROS production, provoke intracellular damage to the oocyte, and affect its competence for embryonic development. To overcome the damage associated with excessive ROS, exogenous antioxidants may be helpful to protect against oxidative stress during in vitro manipulations [7]. Lycopene, a carotenoid, is a natural antioxidant found mainly in ripe tomatoes, watermelon, and pink grapefruit [8]. It is a highly unsaturated straight-chain hydrocarbon with 13 double bonds, 11 of which are conjugated. Lycopene is 10 times more efficient than alpha-tocopherol at scavenging free radicals [9]. Lycopene has demonstrated the potential to scavenge ROS, improve oocyte competence, and enhance embryo development and quality when supplemented to serum-rich IVM medium [10].

Fetal bovine serum (FBS) is commonly added to IVM medium at the rate of 5–20% [11]. The supplementation of IVM media with FBS is important as it contains hormones, vitamins, transport proteins, and growth factors that optimize oocyte maturation. Moreover, it has been reported that FBS has a potent antioxidant activity that neutralizes free radicals and protects oocytes from oxidative stress [12]. For this reason, about 1 million bovine fetuses have to be harvested to obtain about 500,000 L of FBS annually [13], which represents a potential ethical and sanitary concern. Therefore, there is an increasing interest in using serum-free medium [13], and scientists have developed approaches to minimize or replace FBS in cell culture media [14]. We adopted a serum-free maturation medium in which epidermal growth factor (EGF) replaces the utilization of FBS [15]. EGF is a potent cell growth stimulating factor, and its combination with lycopene could serve as a valid alternative to FBS to produce blastocysts with *in vivo*-like gene expression patterns as opposed to embryos produced in serum-rich medium [15,16]. Furthermore, baseline data regarding the antioxidant effects of lycopene alone in biological systems are limited [17]. We hypothesized that the supplementation of antioxidants in a serum-free maturation medium would be effective in obtaining optimal ROS neutralization and the maintenance of equilibrium during IVM and embryo development. This study aimed to assess if the supplementation of serum-free maturation medium with lycopene could reduce ROS generation in matured oocytes and improve the subsequent development, quality, and transcriptomic profile of the embryo, and whether lycopene supplementation during maturation could exert extended protective actions on vitrified-warmed embryos.

## 2. Materials and Methods

### 2.1. Medium Preparation

Tissue culture media (TCM)-199-medium, gentamycin, and kanamycin were obtained from Life Technologies Europe (Ghent, Belgium). Phosphate-buffered saline (PBS) was obtained from Gibco™ Thermo Fisher Scientific (Waltham, MA, USA). All other chemicals not otherwise listed were purchased from Sigma-Aldrich (Diegem, Belgium). All media were filtered before use (0.22 µm filter, Pall Corporation, Ann Arbor, MI, USA).

Lycopene was prepared by dissolving 1 mg of lycopene powder in 5 µL of dimethyl sulfoxide (DMSO) and transferring to 1 mL of distilled water. Thus, the lycopene concentration in the resulting stock solution was 2 mM. The lycopene stock solution was stored at −80 °C. On the day of the experiment, the lycopene stock solution was diluted 20 times in distilled water to obtain a 100 µM work solution. Finally, 1 µL of the lycopene work solution was added to 500 µL of maturation medium to obtain a 0.2 µM final concentration of lycopene.

Menadione was prepared by dissolving 0.034 g of menadione powder in 20 mL of 0.1% ethanol. The solution was stored at 4 °C until further use. On the day of the experiment, 25 µL of menadione solution (10 mM) was added to 75 µL of maturation medium. Finally, 1 µL of the diluted stock solution (2.5 mM) was added into 500 µL of maturation medium to obtain a 5 µM final concentration of menadione.

The decision to supplement 0.2 µM lycopene and 5 µM menadione in our research model was based on the results obtained in previous studies [10,18,19].

### 2.2. In Vitro Embryo Production

*In vitro* embryo production methods were performed as described by Wydooghe et al. [11]. Briefly, bovine ovaries were obtained from the local slaughterhouse and processed within 2 h after collection. Once at the laboratory, the ovaries were disinfected with 96% alcohol and washed three times in warm (37 °C) physiological saline supplemented with kanamycin (50 mg/mL). Cumulus-oocyte complexes (COCs) were aspirated from antral follicles (between 4 and 8 mm in diameter) using an 18-gauge needle attached to a 10 mL syringe. Oocytes aspirated together with follicular fluid were transferred into a 15 mL tube containing 2.5 mL of wash medium (HEPES-TALP). Thereafter, oocytes with homogenous cytoplasm and surrounded by more than three layers of compact cumulus cells were selected and cultured in groups of 60 COCs in 500 µL modified bicarbonate-buffered TCM-199 (supplemented with 50 µg/mL gentamycin and 20 ng/mL EGF) in 5% CO_2_ in air for 22 h at 38.5 °C. The maturation medium was supplemented with 0.2 µM lycopene or antioxidant group, 5 µM menadione or pro-oxidant group, 0.2 µM lycopene plus 5 µM menadione (L + M), or no supplementation (control group).

Frozen-thawed spermatozoa obtained from a proven sire were separated using a 45/90% Percoll^®^ gradient (GE Healthcare Biosciences, Uppsala, Sweden). The sperm pellet was washed in IVF-Tyrode’s albumin–pyruvate–lactate (IVF-TALP) medium, containing bicarbonate-buffered Tyrode solution. The sperm was adjusted to a final concentration of 1x10^6^ spermatozoa/mL using IVF–TALP medium enriched with bovine serum albumin (BSA; 6 mg/mL) and heparin (20 μg/mL). After 21 h of fertilization, the presumed zygotes were vortexed for 3 min in a 15 mL tube containing 2.5 mL HEPES-TALP to remove the zona attached cumulus and sperm cells. The presumed zygotes were washed in HEPES-TALP and subsequently cultured in groups of 25 in 50 µL droplets of synthetic oviductal fluid enriched with non-essential and essential amino acids (SOFaa) and ITS (5 μg/mL insulin; 5 μg/mL transferrin; 5 ng/mL selenium). The droplets were covered with 900 μL paraffin oil (SAGE, Cooper Surgical, Trumbull, CT, USA) and incubated at 38.5 °C in 5% CO_2_, 5% O_2_, and 90% N_2_ for 8 days.

### 2.3. The Effect of Lycopene and Menadione Supplementation to Maturation Medium on Embryo Development and Quality

Lycopene and menadione treatments and embryo production were performed as described above. At 45 h post insemination (hpi), cleavage rate was calculated as the percentage of cleaved embryos out of presumed zygotes. On days 7 and 8, post-insemination (dpi) blastocyst rates were determined as the percentage of blastocysts out of presumed zygotes. In addition, a preliminary experiment was conducted with the solvents used to dissolve lycopene (DMSO) and menadione (ethanol) to exclude the probability of the solvents having any influence on the results.

Differential apoptotic staining was performed to assess the quality of the blastocysts [20]. To do this, day 8 blastocysts were fixed in 4% paraformaldehyde for 20 min at room temperature (RT). The blastocysts were incubated overnight in 0.5% Triton X-100 and 0.05% Tween 20 in PBS at 4 °C. Next, the blastocysts were washed 3 times for 2 min in PBS–BSA. Thereafter, the DNA of the blastomeres was denatured by exposure to 2N HCl for 20 min followed by 100 mM Tris–HCl (pH 8.5) for 10 min at RT. After denaturation and washing (3 times for 2 min in PBS–BSA), the embryos were transferred into blocking solution. The embryos were blocked overnight in 10% goat serum (Invitrogen, Merelbeke, Belgium) and 0.05% Tween 20 in PBS at 4 °C. Negative control embryos were also left in blocking solution. The test embryos were washed and incubated in the ready-to-use primary CDX2 antibody (Biogenex, San Ramon, CA, USA) for 1 day at 4 °C. Next, the test embryos were washed 2 times for 15 min at RT, 500 µL of blocking solution was prepared, and the embryos were incubated overnight at 4 °C in rabbit active caspase-3 antibody (0.768 ng/mL, Cell Signaling Technology, Leiden, The Netherlands). Subsequently, the embryos were washed again (2 times for 15 min) and the test embryos and negative controls were transferred to goat anti-mouse Texas Red antibody (20 ng/mL in blocking solution; Molecular Probes, Merelbeke, Belgium) for 3 days at 4 °C. Next, the embryos were washed twice and incubated in goat anti-rabbit FITC antibody (10 ng/mL in blocking solution; Molecular Probes, Merelbeke, Belgium) for 1 h at RT. Afterward, the embryos were washed two times in two steps of 15 min, and the embryos were moved to nuclear stain with Hoechst 33342 (50 ng/mL in PBS/BSA; Molecular Probes, Merelbeke, Belgium) for 10 min at RT. The evaluation of the embryos was performed after 24 h by fluorescence microscopy (Leica DM 5500B). This staining protocol allowed for the evaluation of the number of trophectoderm (TE) cells, the inner cell mass number (ICM), total cell number TCN (TE + ICM), ICM/TCN ratio, and the total number of apoptotic cells (AC) as well as the ratio of apoptotic cells (ACR; AC/TCN).

### 2.4. The Effect of Lycopene and Menadione Supplementation to Maturation Medium on ROS Production

An experiment was conducted to determine whether menadione would increase ROS production by oocytes during the 22 h of maturation, and whether the supplementation of the maturation medium with lycopene would ameliorate the oxidative stress induced by menadione.

Oocyte collection, maturation, and lycopene and menadione treatments were performed as described earlier. After 22 h of maturation, COCs were denuded of cumulus cells by vortexing the oocytes for 5 min in 2.5 mL HEPES containing 0.08% hyaluronidase. Denuded oocytes were then incubated with maturation medium (with their respective pro- and antioxidant treatment) containing 5 μM CellROX^®^ Green (C10444, Thermo Fisher Scientific, Eugene, Oregon, USA) for 1 h at 38.5 °C and 5% CO_2_ in air. CellROX is a cell-permeant dye that exhibits bright green photo-stable fluorescence upon oxidation and subsequent binding to DNA. After incubation, the oocytes were washed three times for 2 min in 50 μL droplets of PBS containing 1% (*w*/*v*) polyvinylpyrrolidone (PVP), fixed in 4% (*w*/*v*) paraformaldehyde for 20 min, washed three times in PBS–PVP, and mounted in groups of 5 oocytes in a DABCO droplet on siliconized glass slides. The oocytes were examined individually for fluorescence within 3 h after treatment with CellROX using fluorescence microscopy (Leica DM 5500 B) with a green emission filter (I3). Digital images of each oocyte were obtained with the LAS vision software (v.4.1) (Leica DM 5500 B, Wetzlar, Germany). The images were analyzed using the ImageJ software v.1.48 (National Institutes of Health, Bethesda, Rockville, MD, USA). Net fluorescent intensity was calculated by obtaining the average pixel intensity of each oocyte (after manually drawing a boundary around the oocyte) and subtracting the background intensity obtained from a region of the image without the oocyte.

### 2.5. The Effect of Lycopene and Menadione Supplementation to Maturation Media on Vitrified-Warmed Blastocysts 

The media used during the vitrification process were as follows: handling medium (HM; TCM199/Hanks/HEPES supplemented with 20% (*v*/*v*) FBS), equilibration solution (ES; HM supplemented with 7.5% (*v*/*v*) ethylene glycol (EG) and 7.5% (*v*/*v*) DMSO), and vitrification solution (VS; HM supplemented with 15% (*v*/*v*) EG, 15% (*v*/*v*) DMSO, and 0.5 M sucrose).

Embryos were produced, and lycopene and menadione treatments were performed as described before. The blastocysts were vitrified on days 7 and 8 post-insemination (early blastocyst stage), as described by Ortiz-Escribano et al. [21]. Vitrification was performed at RT in two steps in 150 mm Petri dishes. Briefly, two to three blastocysts were placed in one droplet of 70 μL HM, then 3 droplets of the same volume of ES were placed next to this droplet and the HM droplets were mixed with the first droplet of ES. After 2 min, this mixed droplet was mixed with the second ES droplet. After 2 min, the blastocysts were transferred to a fresh, third 70 μL ES droplet for 6 min. The equilibration step was followed immediately by a sequential vitrification step in which five 70 μL droplets of VS were prepared in a 150 mm Petri dish. The embryos were incubated for 5 s in each of the first four droplets (4 droplets × 5 s each) then moved to the last VS droplet for 30 s; finally, the blastocysts surrounded by the lowest possible volume of VS (<1 μL) were placed in a custom-adapted device and then directly transferred to liquid nitrogen (LN_2_). The total time between the first VS droplet and LN_2_ was around 60 s. The custom-adapted device was made of a 0.25 mL straw with its free end cut off horizontally, creating a space that allowed the loading of blastocysts with a minimal volume (<1 μL). A metal wire was inserted at the opposite end to avoid floating the device in the LN_2_ [22]. After 1 month of storage in LN_2_, the custom-made device containing blastocysts was warmed by transferring it quickly into a warm (38.5 °C) HM solution supplemented with 1 M sucrose for 1 min. This was followed by a sequential three-step sucrose wash-out procedure of 0.5 M, 0.25 M, and 0 M sucrose in HM; each step was carried out for 3, 5, and 5 min, respectively. Finally, the blastocysts were washed three times in HM and then cultured in groups of 12 in 50 μL SOF droplets overlaid with parafilm oil at 38.5 °C in 5% CO_2_, 5% O_2_, and 90% N_2_. All treatment groups (control, lycopene, menadione, and L + M) were vitrified and cultured separately. The blastocysts were fixed and differentially stained (as described above) after 48 h of incubation.

### 2.6. RNA Extraction and Sequencing

Total RNA was isolated from eight pools (four control and four lycopene in four replicates) of 10 blastocysts per pool using the RNeasy Micro kit (Qiagen, Germantown, TN, USA) according to the manufacturer’s protocol. The quality and concentration of the RNA samples were examined using an RNA 6000 Pico Chip (Agilent Technologies, Carlsbad, CA, USA) and a Quant-iT RiboGreen RNA assay kit (Life Technologies, Carlsbad, CA, USA), respectively. Transcriptome library preparation was performed by a QIAseq UPX 3′ transcriptome kit (Qiagen) according to the manufacturer’s instructions with 10 PCR cycles. The quality of the library preparation was checked with a high sensitivity DNA chip (Agilent technologies Inc., Santa Clara, CA, USA) and library quantification was performed by qPCR according to the Illumina qPCR quantification protocol (NXTGNT sequencing facility, Ghent, Belgium), followed by the equimolar pooling of libraries based on qPCR. Sequencing was performed on a high throughput Illumina NextSeq 500 flow cell with 20% PhiX spiked in (read 1: 57 cycles; read 2: 27 cycles; and index: 6 cycles).

First, the reads were trimmed with Trim Galore version 0.6.6. (https://github.com/FelixKrueger/TrimGalore, accessed on 07 July 2021) [23] to remove Illumina adapters, poly-A tails, and low-quality bases. The trimmed reads were mapped against the Bos taurus ARS-UCD1.2 reference genome using the STAR software version 2.7.9a [24]. Unique molecular identifiers (UMIs) were used during the sequencing to characterize the expression levels more accurately, and were processed with UMI-tools version 1.1.1 [25]. Finally, the RSEM software, version 1.3.1 [26], was used to generate the count tables. The sequenced data were deposited in the National Center for Biotechnology Information (NCBI) Gene Expression Omnibus (GEO) database (https://www.ncbi.nlm.nih.gov/gds) with accession number GSE192908.

### 2.7. Statistical Analysis and Bioinformatics

The statistical analyses were performed using R-core (version 4.0.4; R Core Team, Vienna, Austria). The oocyte/zygote/embryo was considered as the unit of interest. Generalized mixed effects models were used to test the effects of anti- and pro-oxidant supplementation (control vs. lycopene vs. menadione vs. L + M) on embryo development parameters (cleavage, day 7, and day 8 blastocyst). Similarly, the effects of anti- and pro-oxidant supplementation of the maturation media on oocyte ROS production and blastocyst differential staining parameters were fitted in mixed linear regression models. For all the models the replicate was set as a random effect. Model residuals were assessed using a scatterplot of the studentized residuals for homoscedasticity, a linear predictor for linearity, and a Shapiro–Wilk test for normality. The raw data (continuous variables) were sqrt-, log^2^-, or log^10^-transformed when the residuals of the linear regression models were not normally distributed (*p* < 0.05). For all transformed variables, the residuals were normally distributed (Shapiro–Wilk’s *p* > 0.05). The differences between supplementation groups were assessed using Tukey’s post hoc test. The results are expressed as least squares means and standard errors. For the above mentioned analyses, the R-packages lme4 [27], multcomp [28], and multcompView [29] were utilized. The significance level was set at *p* ≤ 0.05.

Differential gene expression analysis between control and lycopene-treated samples was performed with DESeq2 version 1.32 [30]. DESeq2 was run with the option ‘independentFiltering = TRUE’ to increase detection power. A gene was called differentially expressed (DE) when the Benjamini–Hochberg-adjusted *p*-value was lower than or equal to 0.05 and the absolute value of the log2-fold change was larger than or equal to 1. The heatmap was computed using the pheatmap package based on the normalized counts of the DESeq2 standard method (median ratio method) of the DE genes. To gain further biological insight into these results, an overrepresentation analysis (ORA) and gene set enrichment analysis (GSEA) were applied. Based on a benchmarking study by Geistlinger et al. [31], pathway analysis with down-weighting of overlapping genes (PADOG) was chosen as the method to perform GSEA [32]. Both methods were applied for the gene ontology (GO; http://current.geneontology.org/products/pages/downloads.html, accessed on 23 July 2021) and Kyoto Encyclopedia of Genes and Genomes (KEGG; https://www.genome.jp/kegg/, accessed on 23 July 2021) gene sets. The R package EnrichmentBrowser was used to perform both analyses [33]. Pathways with *p* ≤ 0.05 were considered statistically significant.

## 3. Results

### 3.1. The Effect of the Addition of Solvents (DMSO and Ethanol) on Blastocyst Production and Quality

We evaluated the effects of DMSO (0.000005% final concentration), ethanol (0.00005% final concentration), and DMSO + ethanol supplementation in IVM medium in order to rule out the potential effect of diluents on blastocyst development and quality. Over three replicates (*n* = 479 COCs), none of the developmental (Table 1) nor quality (Table 2) parameters were different (*p* > 0.8) among the diluents or when compared to the control group (no diluents added).

### 3.2. Lycopene during Maturation Improves Blastocyst Production

**Figure 1:**
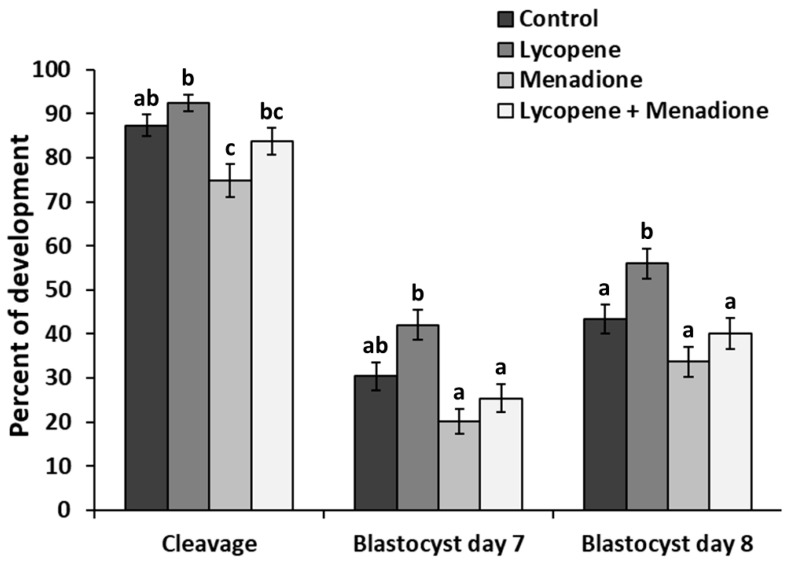
Cleavage, day 7, and day 8 blastocyst rates expressed as a percentage of presumed zygotes. Maturation media was supplemented with lycopene (0.2 μM final concentration), menadione (5 µM final concentration), lycopene + menadione, and no supplementation (control), and standard in vitro fertilization and culture were performed. Different superscripts (a, b, and c) represent statistical differences (*p* < 0.05) between experimental groups. Results are expressed as least square means ± standard errors.

We evaluated the effects of lycopene (0.2 μM final concentration), menadione (5 µM final concentration), and L + M supplementation in IVM medium on cleavage and blastocyst development. The differences in embryo development parameters among the treatment groups (*n* = 806 COCs in 5 replicates) are shown in Figure 1. At 45 hpi, lycopene (92.5 ± 1.97) and L + M (83.8 ± 3.07) supplemented groups did not have a different cleavage rate (*p* > 0.31) when compared to the control group (87.4 ± 2.55). However, a lower cleavage rate (*p* < 0.006) was observed in the menadione group (74.9 ± 3.75) in comparison to the control and lycopene groups. At 7 dpi, no differences in blastocyst rate (*p* > 0.08) were observed in lycopene (42.0 ± 3.4), L + M (25.4 ± 3.17) and menadione (20.2 ± 2.89) groups when compared to the controls (30.4 ± 3.12). At 8 dpi, blastocyst development after oocyte maturation in the lycopene group (56.0 ± 3.45) was higher (*p* < 0.04) than in all the other groups. In day 8 blastocysts, no difference (*p* > 0.18) was found among menadione (33.7 ± 3.40), L + M (40.2 ± 3.57), and the control group (43.3 ± 3.36; Figure 1).

### 3.3. Lycopene during Maturation Improves Blastocyst Quality

**Figure 2:**
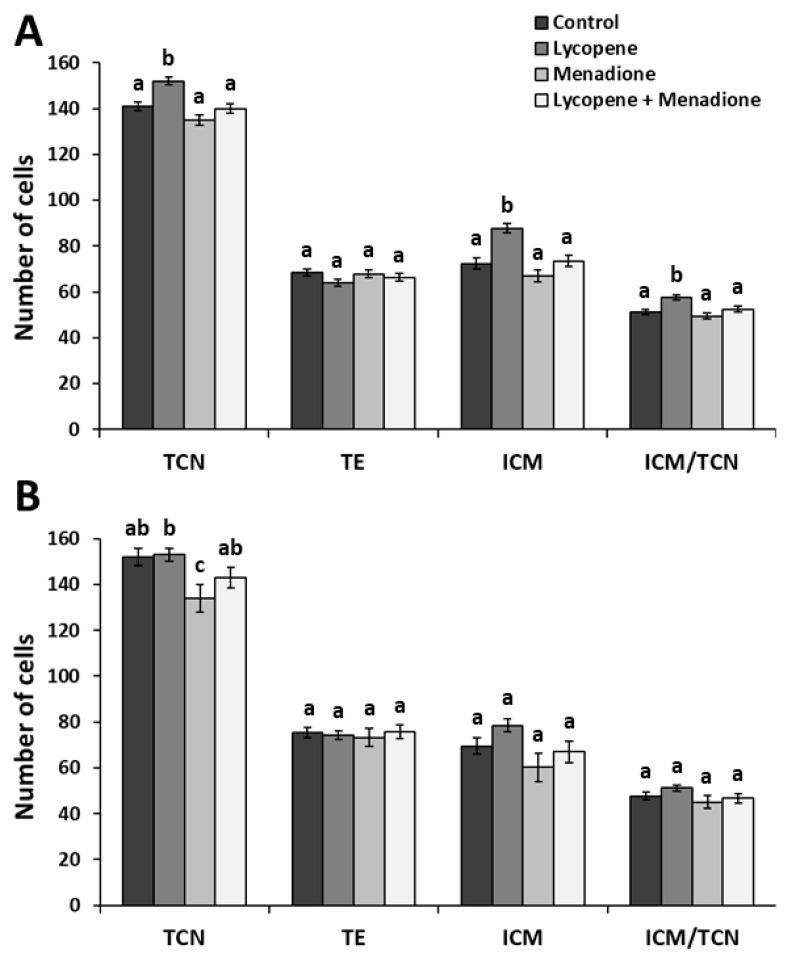
Total cell number (TCN), trophectoderm cells (TE), inner cell mass (ICM), and ICM/TCN of differentially stained day 8 blastocyst before (**A**) and after vitrification-warming (**B**). Maturation media was supplemented with lycopene (0.2 μM final concentration), menadione (5 µM final concentration), lycopene + menadione, and no supplementation (control), and standard in vitro fertilization and culture were performed. Different superscripts (a, b, and c) represent statistical differences (*p* < 0.05) between experimental groups. Results are expressed as least square means ± standard errors.

**Figure 3:**
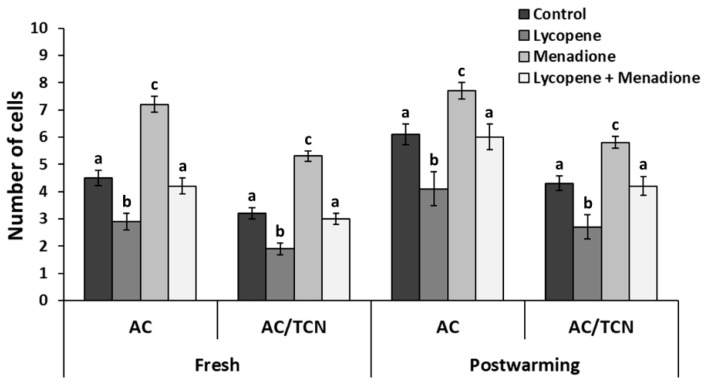
Total apoptotic cells (AC) and AC/total cell number (TCN) of differentially stained day 8 blastocyst before (fresh) and after vitrification and warming (post-warming). Maturation media was supplemented with lycopene (0.2 μM final concentration), menadione (5 µM final concentration), lycopene + menadione, and no supplementation (control), and standard in vitro fertilization and culture were performed. Different superscripts (a, b, and c) represent statistical differences (*p* < 0.05) between experimental groups. Results are expressed as least square means ± standard errors.

We evaluated the effects of lycopene (0.2 μM final concentration), menadione (5 µM final concentration), and L + M supplementation in IVM medium on day 8 blastocyst quality parameters via differential apoptotic immunostaining. The differences in embryo quality for fresh embryos among the treatment groups (*n* = 73 blastocysts in three replicates) are shown in Figure 2A. The TCN and ICM numbers were higher (*p* < 0.001) in the lycopene group (152 ± 1.87 and 87.8 ± 2.23, respectively) than all the other groups. Similarly, the ICM/TCN ratio was higher (*p* < 0.01) in the lycopene group (57.7 ± 1.14) when compared to the other groups. Lower (*p* < 0.004) AC and AC/TCN ratios were recorded in the lycopene group (2.91 ± 2.60 and 1.91 ± 1.89, respectively) compared to all groups (Figure 3). Moreover, higher (*p* < 0.0001) AC and AC/TCN ratios were observed in the menadione group (7.25 ± 3.05 and 5.39 ± 2.21, respectively) compared to all the other groups (Figure 3).

### 3.4. Lycopene during Maturation Reduces Reactive Oxygen Species Production in Matured Oocytes 

**Figure 4:**
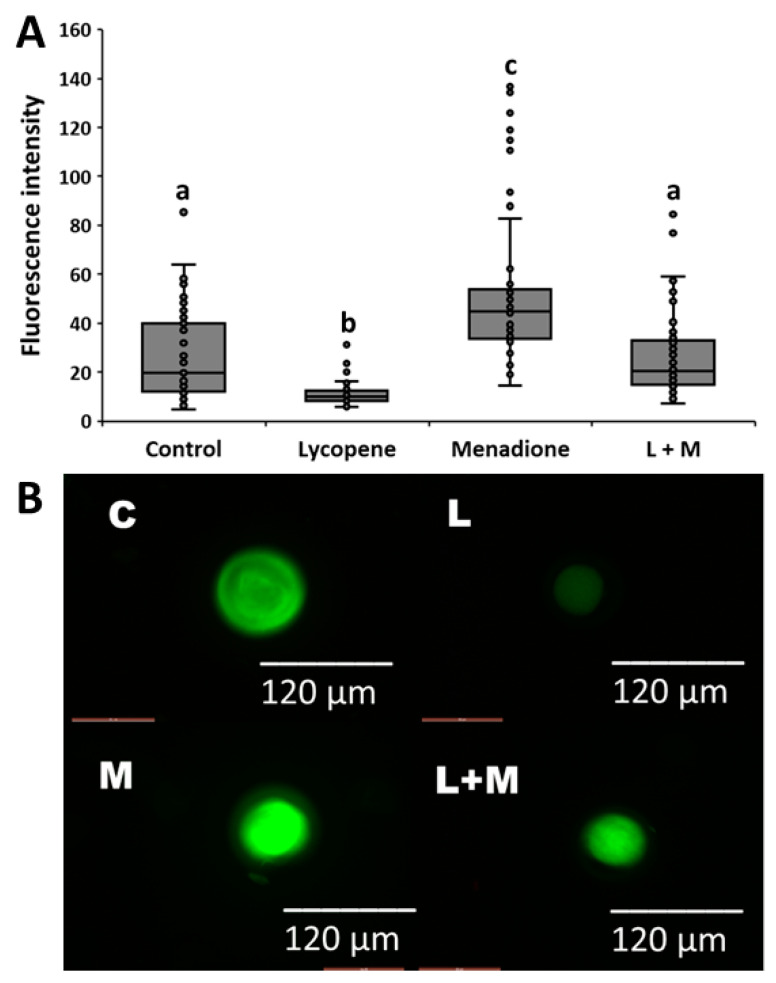
(**A**) Relative fluorescence units of oocytes in the maturation media supplemented with lycopene (L; 0.2 μM final concentration), menadione (M; 5 μM final concentration), lycopene + menadione (L+M), and no supplementation (control; C), and stained with CellROX^®^ to quantify the production of reactive oxygen species. Different superscripts (a, b, and c) represent statistical differences (*p* < 0.05) between experimental groups. (**B**) Representative images of the fluorescence intensities of oocytes matured in the maturation media with L, M, L+M, and no supplementation (C) and stained with CellROX^®^.

We evaluated the effects of lycopene (0.2 μM final concentration), menadione (5 µM final concentration), and L + M supplementation in IVM medium on ROS production in matured oocytes. The differences in fluorescent intensities among the treatment groups (*n* = 273 oocytes in three replicates) are shown in Figure 4. Lower fluorescence intensity values (*p* < 0.0001) were observed in the lycopene group (11.2 ± 2.62) when compared to the other groups (Figure 4). The highest fluorescent intensity values were observed in the menadione group (53.4 ± 2.62), and this value was greater than all the other groups (*p* < 0.0001). The control (26.4 ± 2.62) and L + M (26.1 ± 2.62) groups had similar fluorescent intensities (*p* > 0.99; Figure 4).

### 3.5. Lycopene during Maturation Reduces Post-Warming Apoptosis

We evaluated the extended effects of lycopene (0.2 μM final concentration), menadione (5 µM final concentration), and L + M supplementation in IVM medium on post-warming day 8 blastocyst quality parameters via differential apoptotic immunostaining. A total of 95 early blastocysts (three replicates) were vitrified, and 39 survived after the warming and subsequent 48 h incubation. No differences in survival rate (*p* > 0.22) were found among the experimental groups. The differences in embryo quality for the vitrified embryos among treatment groups (*n* = 39 blastocysts) are shown in Figure 2B. The TCN was not different (*p* > 0.34) among the lycopene (153 ± 2.95), L + M (143 ± 4.59), and control groups (145 ± 3.67). Interestingly, the TCN was lower (*p* < 0.03) in the menadione group (134 ± 6.08) compared to the lycopene group (Figure 2B). Lower (*p* < 0.01) AC and AC/TCN ratios were recorded in the lycopene group (4.12 ± 3.07 and 2.71 ± 2.21, respectively) in comparison to the other groups. Menadione-exposed embryos exhibited more apoptosis, as reflected in the higher values of AC and AC/TCN (7.75 ± 6.33 and 5.82 ± 4.56, respectively) compared to the other groups (*p* < 0.03; Figure 3).

### 3.6. Lycopene during Maturation Changes Blastocyst Transcriptomics

We evaluated the effects of the supplementation of lycopene (0.2 μM final concentration) in IVM medium on day 8 blastocyst transcriptomics compared to control (non-supplemented) blastocysts. Total RNA was extracted from pools of 10 blastocysts in the same stage of development (three hatched, five expanded, and two (normal) blastocysts either for lycopene or control) in four replicates. Based on the striking effects of lycopene supplementation on blastocyst development and quality, we aimed to unravel the transcripts and pathways associated with the obtained results.

**Figure 5:**
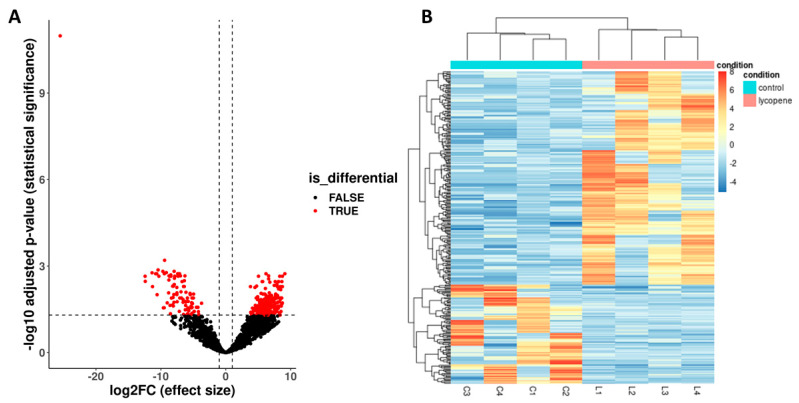
Differentially expressed (DE) genes between lycopene-treated (0.2 μM final concentration) and non-treated (control) day 8 blastocysts. Lycopene treatment was performed during in vitro oocyte maturation. RNA-sequencing was performed from eight pools (four lycopene and four control) of ten blastocysts per pool. (**A**) Volcano plot showing DE genes (*p* < 0.05 and ≥ 1-log2-fold change) between lycopene and control embryos. Two hundred and two genes were upregulated and 94 were downregulated in lycopene versus control. (**B**) Heatmap of the median ratio method of differentially expressed genes. The expression pattern is indicated by a gradient of color from red (upregulation) to blue (downregulation) in lycopene versus control. Branches in the horizontal dendrogram expose perfect clustering in lycopene-treated and control embryos.

Each pool of blastocysts contained an average of 7.75 × 10^6^ million reads. On average, 73.05% of the reads were uniquely mapped to the reference genome by STAR. The gene expression analysis revealed 296 DE genes (Benjamini–Hochberg-adjusted *p* < 0.05 and ≥ 1-log2-fold change) between the lycopene and control groups (Figure 5A). Of the 296 DE genes, 202 genes were upregulated and 94 genes were downregulated in the lycopene group (Figure 5A). The gene expression pattern shows a cluster for both the lycopene and control groups (Figure 5B). 

**Figure 6:**
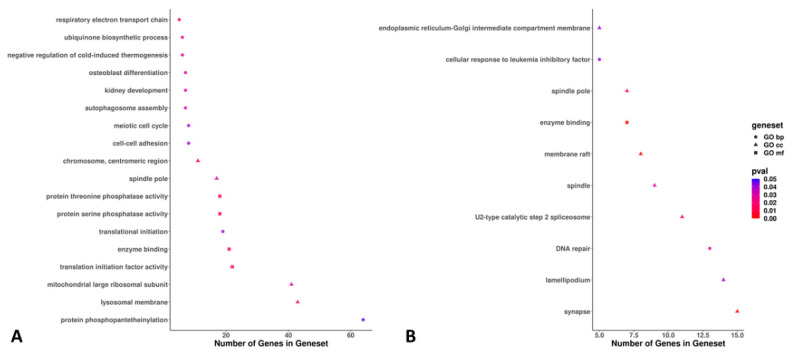
Plots showing the gene/set pathways, number of genes within the set/pathway, and the *p*-values of the differentially expressed gene/set pathways between lycopene-treated (0.2 μM final concentration) and non-treated (control) day 8 blastocysts. Lycopene treatment was performed during in vitro oocyte maturation. RNA-sequencing was performed from eight pools (four lycopene and four control) of ten blastocysts per pool. (**A**) Eighteen gene ontology (GO) terms were found to be differentially expressed using overrepresentation analysis (ORA). (**B**) Ten GO terms were found to be differentially expressed using pathway analysis with down-weighting of overlapping genes (PADOG). Pathways with *p* ≤ 0.05 were considered statistically significant. The gene sets of the differentially expressed GO terms were classified as biological process (BP), cellular component (CC), or molecular function (MF).

**Figure 7:**
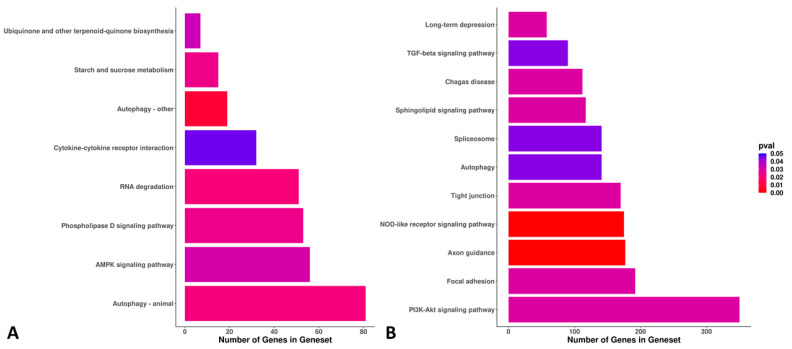
Bar plots showing the gene/set pathways, number of genes within the set/pathway, and the *p*-values of the differentially expressed gene/set pathways between lycopene-treated (0.2 μM final concentration) and non-treated (control) day 8 blastocysts. Lycopene treatment was performed during in vitro oocyte maturation. RNA-sequencing was performed from eight pools (four lycopene and four control) of ten blastocysts per pool. (**A**) Eight Kyoto Encyclopedia of Genes and Genomes (KEGG) pathways were found to be differentially expressed using overrepresentation analysis (ORA). (**B**) Eleven KEGG pathways were found to be differentially expressed using pathway analysis with down-weighting of overlapping genes (PADOG). Pathways with *p* ≤ 0.05 were considered statistically significant.

The 296 DE genes between the lycopene and control groups were associated with 28 significantly expressed GO terms (18 via ORA and 10 via PADOG methods; Figure 6). The most significant GO terms were associated with protein phosphopantetheinylation, the lysosomal membrane, the mitochondrial large ribosomal subunit, synapses, lamellipodium, and DNA repair (Figure 6). For the DE KEGG pathways between the lycopene and control groups (n = 19), we found eight and eleven DE pathways via the ORA and PADOG methods, respectively. The most significant KEGG pathways were autophagy, the AMP-activated protein kinase (AMPK) signaling pathway, the PI3K-Akt signaling pathway, and focal adhesion (Figure 7).

## 4. Discussion

Lycopene supplementation in serum-free maturation medium reduced ROS production in matured oocytes, resulting in a subsequent rise in the blastocyst rate and larger embryos with a lower apoptotic cell ratio than control embryos. Remarkably, lycopene supplementation during oocyte maturation modified the transcriptomic landscape of the resultant blastocyst, including 296 DE genes and multiple DE pathways. Oocytes treated with menadione exhibited higher levels of ROS after maturation and produced blastocysts of lower quality, confirming that our pro-oxidative model was successful. We also showed that when lycopene was supplemented in the presence of menadione, lycopene may help the oocytes to partially restore their developmental capacity (and quality) in a (high) pro-oxidant environment. Lycopene supplementation modestly reduced the AC ratio after the vitrification and warming of the blastocysts. The IVM environment per se is a milieu rich in ROS. Therefore, we encourage the routine supplementation of antioxidants, such as lycopene, during maturation to improve the blastocyst yield and quality in IVEP. 

It is evident that oxidative stress during IVM is associated with abnormal embryo development and quality, and high oxygen tension during IVM may be a key factor responsible for ROS generation [34]. Thus, it is plausible to see an increasing number of reports emphasizing the beneficial role of the antioxidant supplementation of maturation medium to overcome oxidative stress [35]. Multiple studies reported an increase in blastocyst rate when the IVM medium was supplemented with different antioxidants, such as nobiletin [36], quercetin [37], vitamin C [38], and resveratrol [39]. Chowdhury et al. [10] were the first to evaluate the effect of lycopene in IVM in a bovine model. Due to its unique structure of conjugated double bonds, lycopene has a high affinity to and rapidly quenches superoxide (O_2_) and other free radical anions typically abundant during in vitro maturation. Chowdhury et al. [10] evaluated several doses of lycopene ranging from 0.1 µM to 1.5 µM in IVM serum-rich medium, and the highest blastocyst rate was obtained in the presence of 0.2 µM lycopene (35% in 0.2 µM lycopene vs. 28% in control). Nonetheless, in all those studies, IVM was also supplemented with FBS, which contains a plethora of antioxidants in unknown and variable (according to the batch) concentrations [12]. Fortunately, serum-free media formulations with the addition of EGF or BSA are increasing worldwide, and some formulations result in similar or even better-matured oocytes with higher developmental competence than those from serum-supplemented medium [40]. Thus, in a recent study, under serum-free conditions, we used 0.2 µM lycopene supplementation and obtained a day 8 blastocyst rate of 55% (control was 44%) with 10% more TCN than the control group [18]. However, in that study, lycopene supplementation could not restore embryo production and quality under heat-shock conditions (oocytes matured at 40.5 °C). In the present study, we were able to replicate the results of 0.2 µM lycopene supplementation on improved blastocyst production (56% in lycopene vs. 43% in control) and quality (8% more TCN and a lower apoptotic cell ratio in lycopene than control). However, under pro-oxidant conditions (supplementation with menadione), 0.2 µM lycopene seems to be insufficient to improve blastocyst production.

The quality of the embryo has been found to be essential to achieve a successful pregnancy after IVEP [41]. Blastocyst expansion, ICM, and TE are parameters used to determine quality along with a normal, balanced morphological structure, cell number, and cell distribution [42]. The critical role of TE in mediating correct embryo implantation is well established. [43]. Furthermore, ICM was shown to promote the proliferation of TE cells in human embryos [43]. The TCN of the mammalian embryo, with concurrent details on differential cell allocation and the presence or absence of AC, provide detailed information regarding embryo quality [44], even more so than quantitative gene expression in the embryo, which can be influenced by any short impulse. Interestingly, the higher TCN, ICM, and ICM/TCN ratio and lower AC number recorded in the embryos exposed to lycopene during IVM could be associated with the ability of lycopene to inhibit certain pro-apoptotic pathways, such as the binding activity of NF-κB and the expression of the NF-κB target gene MMP-9 [45]. Such inhibition is mediated by the downregulation of kappa B (IκB) phosphorylation, NF-κB expression, and NF-κB p65 subunit translocation from the cytosol to the nucleus [45].

Our results suggest that high levels of ROS are present in serum-free maturation medium, supported by the high intensity values of ROS (compared to lycopene) recorded in the control matured oocytes. ROS are generated through the leakage of electrons from the inner mitochondrial membrane during oxidative phosphorylation [46]. Many environmental factors (oxygen tension, the amount of UV light, cellular manipulations, and centrifugation) may expose sperm, oocytes, and embryos to ROS during IVEP procedures, which is believed to influence the blastocyst production and quality [47]. This (high) generation of ROS might oxidize vital cellular biomolecules, such as carbohydrates, lipids, proteins, and DNA; the oxidation of these molecules leads to apoptosis, DNA damage, and genetic instability [48]. Three different approaches are suggested for the assessment of oxidative stress: (1) the direct measurement of ROS levels, (2) the detection of the resulting oxidative damage to biomolecules (DNA, lipids, and proteins), and (3) the determination of antioxidant status (enzymatic antioxidant activities, nonenzymatic antioxidant levels, or total antioxidant capacity). However, direct ROS determination seems to be the most valuable method [49]. Our results suggest a lower ROS generation in the lycopene-supplemented group. Thus, we can make a plea for the routine supplementation of antioxidants into serum-free IVM medium because oocyte maturation is typically performed at high atmospheric oxygen tensions, increasing ROS production. In our hands, maturation at 21% O_2_ has produced higher meiotic maturation and better blastocyst development than maturation at 5% O_2_ (data not shown).

Embryo cryopreservation can significantly increase ROS production and subsequent cell damage [50]. Although the vitrification process was designed to reduce cryoinjury, the total elimination of cell damage cannot not be guaranteed [51]. Oxidative stress due to cryopreservation may be generated through different mechanisms, such as osmotic stress and increased oxidative metabolism. In somatic cells, osmotic stress activates the generation of superoxide anions caused by the activation of NADPH oxidase [52]. These results can be taken as evidence that cryopreservation induces damage to the structures of organelles [53]. Blastocysts in the lycopene group had a lower AC number and ratio after vitrification/warming than all the other experimental groups. Thus, the present study indicated that the supplementation of maturation medium with lycopene displayed an extended antioxidant activity which was still present in the embryo during vitrification/warming, likely by reducing the oxidative stress caused by cryopreservation.

Remarkably, the RNA-sequencing results of the present study showed that lycopene treatment during IVM reduced the expression of CASP3 and BAX and increased the expression of BCL2 in the blastocyst compared to the control. This is important because all apoptotic pathways appear to terminate in activating the caspase family of proteases, which is regulated by the BCL2 family of proteins [54]. Similarly, the pro-apoptotic gene BAX can promote the release of cytochrome c from the mitochondria into the cytoplasm, leading to apoptosis [55]. Interestingly, the BCL2 gene codes for an integral outer mitochondrial membrane protein that blocks apoptotic death [56]. Thus, our RNA-sequencing results (at the individual gene expression level) are concordant with the phenotype of blastocysts with a lower AC value and AC/TCN ratio in the lycopene group compared to the control. Furthermore, the downregulation of NFKB1 and IKBKB gene expression in the lycopene group is also critical in regulating apoptosis. The activity of NFKB1 (a transcription factor that can induce inflammation, leading to a rapid increase in ROS concentration and cell apoptosis) is tightly regulated by the inhibitor of kappa-B (IKB; includes IKBKa, IKBKB, and IKBKƐ) proteins and the IkB kinase complex [57], which intercept NFKB1 dimers in the cytoplasm [58]. Although NFKB1 is activated in the nucleus, its inhibition can be brought about indirectly by inhibiting IkB in the cytoplasm [59]. Finally, we postulate a potential anti-inflammatory property of lycopene (directly or indirectly) via the downregulation of COX2 (7-fold) in lycopene compared to the control group.

In the current study, several GO and KEGG pathways were DE between the lycopene and control groups. One of the upregulated pathways in lycopene was autophagy, an essential process that maintains cellular homeostasis and functions [60]. In the same line, the DNA repair pathway was upregulated in the lycopene group, indicating stimulation to maintain genetic stability and integrity when the embryos were exposed to endogenous or exogenous DNA-damaging agents, such as ROS [61]. The maintenance of active cellular growth was also demonstrated by the upregulation of the translational initiation and translational initiation factor activity pathways in the lycopene group. Moreover, phospholipase D was also upregulated in the lycopene group. The phospholipase D pathway regulates intercellular signaling and metabolic pathways, particularly in cells under stress conditions [62]. Likewise, other anabolic and intra- and inter-cellular signaling pathways such as the meiotic cell cycle, cell–cell adhesion, AMPK signaling, starch and sucrose metabolism, and other pathways were all upregulated in the lycopene group. Interestingly, the sphingolipid signaling pathway was also upregulated in the lycopene group. The sphingolipid signaling pathway is an anti-apoptotic pathway associated with cell membrane stability [63]. Remarkably, the ubiquinone and other terpenoid-quinone biosynthesis pathways were upregulated in the lycopene group. Lycopene is a tetraterpene (from the terpenes family); hence, this study confirms that DMSO is an efficient solvent for lycopene delivery to the oocyte and also demonstrates that lycopene supplementation during oocyte maturation is still reflected in the gene expression of more advanced embryonic stages, for example, in blastocysts. In addition, the cytokine pathway was also upregulated in the lycopene group. Cytokines are soluble extracellular proteins or glycoproteins that are crucial intercellular regulators and mobilizers of cells engaged in innate and adaptive inflammatory host defenses, cell growth, differentiation, cell death, angiogenesis, and the development and repair processes aimed at the restoration of homeostasis [64].

## 5. Conclusions

Lycopene supplementation during IVM significantly improved embryo development, blastocyst quality, and cryo-tolerance and reduced ROS generation. Furthermore, lycopene supplementation changed the transcriptomic landscape of the resultant embryos by upregulating pathways associated with cellular function, metabolism, DNA repair, and anti-apoptosis. When oocytes were co-incubated with menadione, the detrimental effects of ROS were evident; when lycopene was used to counteract such effects, the quality of the embryos tended to improve. This may suggest that the concentration of lycopene needs to be adjusted in the presence of pro-oxidants to improve further developmental capacity. These observations suggest that the supplementation of serum-free maturation medium with lycopene could be useful in managing oxidative stress, even further downstream in the IVEP process. Further studies are therefore encouraged to determine what concentration of lycopene is effective when oocytes are exposed to pro-oxidant conditions. Likewise, different concentrations of lycopene in serum-free IVM and IVC media need to be investigated to determine at what level of IVEP lycopene is more beneficial.

## Figures and Tables

**Table 1:** Cleavage, day 7, and day 8 blastocyst rates expressed as a percentage of presumed zygotes. Maturation media was supplemented with diluents DMSO (0.000005% final concentration), ethanol (0.00005% final concentration), DMSO + ethanol, and no supplementation (control), and standard in vitro fertilization and culture were performed. No differences were found among the experimental groups (*p* > 0.8). Results are expressed as least square means ± standard errors.

Treatment	No. of COCs	Embryo Development		
		Cleavage	Blastocyst Day 7	Blastocyst Day 8
Control	124	85.5 ± 2.5	35.7 ± 4.9	39.7 ± 4.5
DMSO	120	86.7 ± 1.9	31.7 ± 4.8	38.1 ± 4.4
Ethanol	114	81.3 ± 3.7	29.1 ± 4.7	37.5 ± 4.5
DMSO + ethanol	121	83.3 ± 3.1	29.9 ± 4.1	36.8 ± 4.7

**Table 2:** Total cell number (TCN), trophectoderm cells (TE), inner cell mass (ICM), and apoptotic cells (AC) of day 8 differentially stained blastocysts. Maturation media was supplemented with diluents DMSO (0.000005% final concentration), ethanol (0.00005% final concentration), DMSO + ethanol, and no supplementation (control), and standard in vitro fertilization and culture were performed. No differences were found among the experimental groups (*p* > 0.8). Results are expressed as least square means ± standard errors.

Treatment	No. of Blastocysts	Cell Numbers			
		TCN	ICM	TE	AC
Control	45	143 ± 1.5	65.7 ± 1.3	78.4 ± 0.8	3.0 ± 0.1
DMSO	43	144 ± 1.5	67.5 ± 1.4	76.7 ± 0.8	3.2 ± 0.1
Ethanol	41	137 ± 1.4	64.4 ± 1.3	72.8 ± 0.7	2.9 ± 0.1
DMSO + ethanol	42	136 ± 1.6	61.1 ± 1.4	74.7 ± 0.8	3.6 ± 0.1

## Data Availability

Embryo production data is contained within the article. Sequenced data were deposited in the National Center for Biotechnology Information (NCBI) Gene Expression Omnibus (GEO) database (https://www.ncbi.nlm.nih.gov/gds) with accession number GSE192908.

## References

[1] Ahmad G., Almasry M., Dhillon A.S., Abuayyash M.M., Kothandaraman N., Cakar Z. (2017). Overview and sources of reactive oxygen species (ROS) in the reproductive system. Oxidative Stress in Human Reproduction: Shedding Light on a Complicated Phenomenon.

[2] Bedaiwy M.A., Falcone T., Mohamed M.S., Aleem A.A.N., Sharma R.K., Worley S.E., Thornton J., Agarwal A. (2004). Differential growth of human embryos in vitro: Role of reactive oxygen species. Fertil. Steril..

[3] Pasqualotto E.B., Agarwal A., Sharma R.K., Izzo V.M., Pinotti J.A., Joshi N.J., Rose B.I. (2004). Effect of oxidative stress in follicular fluid on the outcome of assisted reproductive procedures. Fertil. Steril..

[4] Campos Petean C., Ferriani R.A., dos Reis R.M., Dias de Moura M., Jordão A.A., Andrea de Albuquerque Salles Navarro P. (2008). Lipid peroxidation and vitamin E in serum and follicular fluid of infertile women with peritoneal endometriosis submitted to controlled ovarian hyperstimulation: A pilot study. Fertil. Steril..

[5] Pavani K.C., Hendrix A., Van Den Broeck W., Couck L., Szymanska K., Lin X., De Koster J., Van Soom A., Leemans B. (2019). Isolation and characterization of functionally active extracellular vesicles from culture medium conditioned by bovine embryos in vitro. Int. J. Mol. Sci..

[6] Gosden R.G., Byatt-Smith J.G. (1986). Oxygen concentration gradient across the ovarian foUicular epithelium: Model, predictions and implications. Human Reproduct..

[7] Young I.S., Woodside J.V. (2001). Antioxidants in health and disease. J. Clin. Pathol..

[8] Bramley P.M. (2000). Molecules of Interest Is lycopene benecial to human health?. Phytochemistry.

[9] Rao L.G., Guns E., Rao A.V. (2003). Lycopene: Its role in human health and disease. Agro Food.

[10] Chowdhury M.M.R., Choi B.H., Khan I., Lee K.L., Mesalam A., Song S.H., Xu L., Joo M.D., Afrin F., Kong I.K. (2017). Supplementation of lycopene in maturation media improves bovine embryo quality in vitro. Theriogenology.

[11] Wydooghe E., Heras S., Dewulf J., Piepers S., Van Den Abbeel E., De Sutter P., Vandaele L., Van Soom A. (2014). Replacing serum in culture medium with albumin and insulin, transferrin and selenium is the key to successful bovine embryo development in individual culture. Reprod. Fertil. Dev..

[12] Van Der Valk J., Mellor D., Brands R., Fischer R., Gruber F., Gstraunthaler G. (2004). The humane collection of fetal bovine serum and possibilities for serum-free cell and tissue culture. Toxicol. Vitro.

[13] van der Valk J., Bieback K., Buta C., Cochrane B., Dirks W.G., Fu J., Hickman J.J., Hohensee C., Kolar R., Liebsch M. (2018). Fetal Bovine Serum (FBS): Past-Present-Future. ALTEX.

[14] Jochems C.E.A., Van Der Valk J.B.F., Stafleu F.R., Baumans V. (2002). The Use of Fetal Bovine Serum: Ethical or Scientific Problem?. Alternat. Lab. Animals.

[15] Lonergan P., Carolan C., Van Langendonckt A., Donnay I., Khatir H., Mermillod P., Vtrinaire U.C.L.U. (1996). Role of Epidermal Growth Factor in Bovine Oocyte Maturation and Preimplantation Embryo Development In Vitro. Biol. Reproduct..

[16] Heras S., De Coninck D.I.M., Van Poucke M., Goossens K., Bogado Pascottini O., Van Nieuwerburgh F., Deforce D., De Sutter P., Leroy J.L.M.R., Gutierrez-Adan A. (2016). Suboptimal culture conditions induce more deviations in gene expression in male than female bovine blastocysts. BMC Genom..

[17] Kelkel M., Schumacher M., Dicato M., Diederich M. (2011). Antioxidant and anti-proliferative properties of lycopene. Free Radical Res..

[18] Residiwati G., Azari-Dolatabad N., Tuska H.S.A., Sidi S., Van Damme P., Benedetti C., Montoro A.F., Luceno N.L., Budiono, Pavani K.C. (2021). Effect of lycopene supplementation to bovine oocytes exposed to heat shock during in vitro maturation. Theriogenology.

[19] Cavallari F.D.C., Leal C.L.V., Zvi R., Hansen P.J. (2019). Effects of melatonin on production of reactive oxygen species and developmental competence of bovine oocytes exposed to heat shock and oxidative stress during in vitro maturation. Zygote.

[20] Wydooghe E., Vandaele L., Beek J., Favoreel H., Heindryckx B., De Sutter P., Van Soom A. (2011). Differential apoptotic staining of mammalian blastocysts based on double immunofluorescent CDX2 and active caspase-3 staining. Anal. Biochem..

[21] Ortiz-Escribano N., Szymańska K.J., Bol M., Vandenberghe L., Decrock E., Van Poucke M., Peelman L., Van den Abbeel E., Van Soom A., Leybaert L. (2017). Blocking connexin channels improves embryo development of vitrified bovine blastocysts. Biol. Reprod..

[22] Ortiz-Escribano N., Bogado Pascottini O., Woelders H., Vandenberghe L., De Schauwer C., Govaere J., Van den Abbeel E., Vullers T., Ververs C., Roels K. (2018). An improved vitrification protocol for equine immature oocytes, resulting in a first live foal. Equine Vet. J..

[23] Krueger F. (2015). Trim Galore: A Wrapper Tool around Cutadapt and FastQC to Consistently Apply Quality and Adapter Trimming to FastQ Files. https://www.bioinformatics.babraham.ac.uk/projects/trim_galore.

[24] Dobin A., Davis C.A., Schlesinger F., Drenkow J., Zaleski C., Jha S., Batut P., Chaisson M., Gingeras T.R. (2013). STAR: Ultrafast universal RNA-seq aligner. Bioinform..

[25] Smith T., Heger A., Sudbery I. (2017). UMI-tools: Modeling sequencing errors in Unique Molecular Identifiers to improve quantification accuracy. Genome Res..

[26] Li B., Dewey C.N. (2011). RSEM: Accurate transcript quantification from RNA-Seq data with or without a reference genome. BMC Bioinform..

[27] Bates D., Mächler M., Bolker B., Walker S. (2014). Fitting Linear Mixed-Effects Models using lme4. arXiv.

[28] Hothorn T., Bretz F., Westfall P. (2008). Simultaneous inference in general parametric models. Biometrical J..

[29] Graves H.P.S. (2019). Package “multcompView” Title Visualizations of Paired Comparisons. https://cran.r-project.org/web/packages/multcompView/index.html.

[30] Love M.I., Huber W., Anders S. (2014). Moderated estimation of fold change and dispersion for RNA-seq data with DESeq2. Genome Biol..

[31] Geistlinger L., Csaba G., Santarelli M., Ramos M., Schiffer L., Turaga N., Law C., Davis S., Carey V., Morgan M. (2021). Toward a gold standard for benchmarking gene set enrichment analysis. Brief. Bioinform..

[32] Tarca A.L., Draghici S., Bhatti G., Romero R. (2012). Down-weighting overlapping genes improves gene set analysis. BMC Bioinform..

[33] Geistlinger L., Csaba G., Zimmer R. (2016). Bioconductor’s EnrichmentBrowser: Seamless navigation through combined results of set- & network-based enrichment analysis. BMC Bioinform..

[34] Agarwal A., Allamaneni S.S.R., Nallella K.P., George A.T., Mascha E. (2005). Correlation of reactive oxygen species levels with the fertilization rate after in vitro fertilization: A qualified meta-analysis. Fertil. Steril..

[35] Sovernigo T.C., Adona P.R., Monzani P.S., Guemra S., Barros F.D.A., Lopes F.G., Leal C.L.V. (2017). Effects of supplementation of medium with different antioxidants during in vitro maturation of bovine oocytes on subsequent embryo production. Reprod. Domest. Anim..

[36] Cajas Y.N., Cañón-Beltrán K., de Guevara M.L., Millán de la Blanca M.G., Ramos-Ibeas P., Gutiérrez-Adán A., Rizos D., González E.M. (2020). Antioxidant nobiletin enhances oocyte maturation and subsequent embryo development and quality. Int. J. Mol. Sci..

[37] Guemra S., Monzani P.S., Santos E.S., Zanin R., Ohashi O.M., Miranda M.S., Adona P.R. (2013). Maturação in vitro de oócitos bovinos em meios suplementados com quercetina e seu efeito sobre o desenvolvimento embrionário. Arq. Bras. Med. Veterinária e Zootec..

[38] Kere M., Siriboon C., Lo N.W., Nguyen N.T., Ju J.C. (2013). Ascorbic acid improves the developmental competence of porcine oocytes after parthenogenetic activation and somatic cell nuclear transplantation. J. Reprod. Dev..

[39] Kwak S.S., Cheong S.A., Jeon Y., Lee E., Choi K.C., Jeung E.B., Hyun S.H. (2012). The effects of resveratrol on porcine oocyte in vitro maturation and subsequent embryonic development after parthenogenetic activation and in vitro fertilization. Theriogenology.

[40] Stroebech L., Mazzoni G., Pedersen H.S., Freude K.K., Kadarmideen H.N., Callesen H., Hyttel P. (2015). In vitro production of bovine embryos: Revisiting oocyte development and application of systems biology. Anim. Reprod..

[41] Gardner D.K., Lane M. (2000). Embryo culture system. Handbook of In Vitro Fertilization.

[42] Papanikolaou E.G., Kolibianakis E.M., Tournaye H., Venetis C.A., Fatemi H., Tarlatzis B., Devroey P. (2008). Live birth rates after transfer of equal number of blastocysts or cleavage-stage embryos in IVF. A systematic review and meta-analysis. Hum. Reprod..

[43] Thompson S.M., Onwubalili N., Brown K., Jindal S.K., McGovern P.G. (2013). Blastocyst expansion score and trophectoderm morphology strongly predict successful clinical pregnancy and live birth following elective single embryo blastocyst transfer (eSET): A national study. J. Assist. Reprod. Genet..

[44] Kafi M. (2005). Differential staining combined with TUNEL labelling to detect apoptosis in preimplantation bovine embryos Related papers. Reproduct. Biomed. Online.

[45] Hung C.F., Huang T.F., Chen B.H., Shieh J.M., Wu P.H., Wu W. (2008). Bin Lycopene inhibits TNF-α-induced endothelial ICAM-1 expression and monocyte-endothelial adhesion. Eur. J. Pharmacol..

[46] Holley A.K., Bakthavatchalu V., Velez-Roman J.M., Clair D.K. (2011). Manganese superoxide dismutase: Guardian of the powerhouse. Int. J. Mol. Sci..

[47] Smith G.D., Takayama S. (2017). Application of microfluidic technologies to human assisted reproduction. Mol. Hum. Reprod..

[48] Sharma A., Gupta P., Prabhakar P.K. (2019). Endogenous Repair System of Oxidative Damage of DNA. Curr. Chem. Biol..

[49] Katerji M., Filippova M., Duerksen-Hughes P. (2019). Approaches and methods to measure oxidative stress in clinical samples: Research applications in the cancer field. Oxid. Med. Cell. Longev..

[50] Dowling D.K., Simmons L.W. (2009). Reactive oxygen species as universal constraints in life-history evolution. Proc. R. Soc. B Biol. Sci..

[51] Gardner D.K., Sheehan C.B., Rienzi L., Katz-Jaffe M., Larman M.G. (2007). Analysis of oocyte physiology to improve cryopreservation procedures. Theriogenology.

[52] Agarwal A., Virk G., Ong C., du Plessis S.S. (2014). Effect of Oxidative Stress on Male Reproduction. World J. Mens. Health.

[53] Tatone C., Di Emidio G., Vento M., Ciriminna R., Artini P.G. (2010). Cryopreservation and oxidative stress in reproductive cells. Gynecol. Endocrinol..

[54] Exley G.E., Tang C., Mcelhinny A.S., Warner C.M. (1999). Expression of Caspase and BCL-2 Apoptotic Family Members in Mouse Preimplantation Embryos. Biol. Reproduct..

[55] Zhao X.M., Hao H.S., Du W.H., Zhao S.J., Wang H.Y., Wang N., Wang D., Liu Y., Qin T., Zhu H. (2016). Bin Melatonin inhibits apoptosis and improves the developmental potential of vitrified bovine oocytes. J. Pineal Res..

[56] Youle R.J., Strasser A. (2008). The BCL-2 protein family: Opposing activities that mediate cell death. Nat. Rev. Mol. Cell Biol..

[57] Gilmore T.D. (2006). Introduction to NF-κB: Players, pathways, perspectives. Oncogene.

[58] Karin M., Cao Y., Greten F.R., Li Z.W. (2002). NF-κB in cancer: From innocent bystander to major culprit. Nat. Rev. Cancer.

[59] Serasanambati M., Chilakapati S.R. (2016). Function of Nuclear Factor Kappa B (NF-kB) in Human Diseases—A Review. South Indian J. Biol. Sci..

[60] Jing K., Lim K. (2012). Why is autophagy important in human diseases?. Exp. Mol. Med..

[61] Li L.Y., Guan Y.D., Chen X.S., Yang J.M., Cheng Y. (2021). DNA Repair Pathways in Cancer Therapy and Resistance. Front. Pharmacol..

[62] Bruntz R.C., Lindsley C.W., Brown H.A. (2014). Phospholipase D signaling pathways and phosphatidic acid as therapeutic targets in cancer. Pharmacol. Rev..

[63] Hla T., Dannenberg A.J. (2012). Sphingolipid signaling in metabolic disorders. Cell Metab..

[64] Turner M.D., Nedjai B., Hurst T., Pennington D.J. (2014). Cytokines and chemokines: At the crossroads of cell signalling and inflammatory disease. Biochim. Biophys. Acta Mol. Cell Res..

